# When would orthopaedic surgeons perform arthroplasty for a femoral neck fracture in an older adult?

**DOI:** 10.1007/s00590-025-04412-3

**Published:** 2025-07-11

**Authors:** Jose Manuel De Maria Prieto, Joseph T. Patterson, Olivia Paige Szasz, Sofia Bzovsky, Ernesto Guerra-Farfán, Daniel Axelrod, Soroush Shabani, Gerard P. Slobogean, Sheila Sprague

**Affiliations:** 1 Division of Orthopaedic Surgery, Department of Surgery, McMaster University, Hamilton, Canada; 2 Keck School of Medicine of the University of Southern California, Los Angeles, United States; 3 Department of Traumatology, Orthopaedic Surgery and Emergency, Hospital Vall d’Hebrón, Barcelona, Spain; 4 Center for Orthopaedic Injury Research and Innovation, Department of Orthopaedics, University of Maryland School of Medicine, R Adams Cowley Shock Trauma Center, Baltimore, United States; 5 Department of Health Research Methods, Evidence, and Impact, McMaster University, Hamilton, Canada

**Keywords:** Femoral neck fractures, Minimal displacement, Internal fixation, Arthroplasty

## Abstract

**Purpose:**

Minimally displaced femoral neck fractures (FNFs) in older adults have traditionally been managed with internal fixation (IF). However, emerging evidence suggests arthroplasty may provide better outcomes. We sought to determine surgeons’ current practice patterns and determine which patient and fracture characteristics lead them to prefer arthroplasty.

**Methods:**

We developed a survey to assess the influence of fracture and patient characteristics on orthopaedic surgeons’ choice to treat FNFs in older adults with arthroplasty. We electronically distributed the survey to members of professional associations and our research network.

**Results:**

Among 155 orthopaedic surgeons (response rate 25%), 74% agreed that deciding between IF and arthroplasty is difficult for certain minimally displaced FNFs cases and 36% reported performing arthroplasty for at least half of minimally displaced FNFs. Surgeons reported they would perform arthroplasty for a minimally displaced FNF with posterior tilt of 20° (69%) or 30° (94%), varus angulation (88%), or a neck-shaft angle > 160° (70%). Age (83%), mobility (76%), and osteoporosis (62%) influenced surgeons’ treatment preferences. Preference for arthroplasty was significantly associated with annual volume of minimally displaced FNFs (*p* = 0.033), but not years in practice (*p* = 0.065). Seventy-nine per cent agreed that a randomized trial is needed to determine the best clinical practice for minimally displaced FNFs.

**Conclusions:**

In contrast to existing guidelines and practice trends, over one-third of orthopaedic surgeons who responded to the questionnaire would routinely treat minimally displaced FNFs with arthroplasty. The variation between surgeon’s current practices demonstrates the need for a high-quality randomized trial.

**Supplementary Information:**

The online version contains supplementary material available at 10.1007/s00590-025-04412-3.

## Introduction

Fragility hip fractures affect approximately 300,000 older adults annually in the USA, with global incidence expected to exceed 6 million per year by 2050 [1, 2]. Femoral neck fractures (FNFs) account for nearly half of hip fractures in older adults, with an average of 20%−30% being minimally displaced [3, 4]. FNFs are associated with high rates of complications, long-term disability, and mortality, with mortality rates of up to 10% during the first month after surgery and up to 36% during the first year after surgery [5–8].

The two primary surgical treatments for FNFs are hip arthroplasty and IF [9]. Arthroplasty is the standard of care for displaced FNFs in older adults [10–12]. Minimally displaced FNFs have traditionally been treated with IF as it is less invasive and has a shorter surgical time [13–15]. In many parts of the world, IF remains the preferred treatment for minimally displaced FNFs [16].

However, emerging evidence suggests arthroplasty for minimally displaced FNF improves patient outcomes with lower risk of major reoperations, complications, pain, and mortality, and better hip function and quality of life compared to IF [13, 17–21]. Previous research has found that sex (females), age (over 50 years), smoking, diabetes, ASA grade, and posterior tilt ≥ 20° are associated with an increased risk of failure of fracture fixation [22–24]. In response, some orthopaedic surgeons have begun to question whether IF is the best treatment for minimally displaced FNFs and some are changing their practice [25].

Minimally displaced FNFs are currently defined as Type 1 and Type 2 fractures according to the Garden classification system [26, 27]. However, the Garden classification has limitations for quantitatively describing displacement. The Garden classification only considers fracture angulation and separation in the coronal plane. The angulation assessment does not scale severity. Importantly, Garden’s scheme does not assess posterior tilt in the axial plane, which emerging evidence suggests is strongly associated with increased risk of failure following IF. This survey aimed to gain an understanding of surgeons’ current practice patterns in managing minimally displaced FNFs and define the bounds of equipoise for sufficient displacement to perform arthroplasty.

## Methods

### Survey development

We developed an electronic survey on SurveyMonkey to determine the treatment preferences for minimally displaced FNFs and to assess the influence of fracture and patient characteristics on orthopaedic surgeons’ choice to treat minimally displaced FNFs with IF or arthroplasty using clinical vignettes. The questionnaire was developed with input from a quantitative methodologist, clinical trials expert [28], referencing similar surveys [29, 30], and relevant literature on minimally displaced FNF [13, 17–21], as well as input from key stakeholders, including orthopaedic surgeons with and without fellowship training in arthroplasty and trauma. Orthopaedic surgeons with varying experience managing minimally displaced FNF were included in the study to accurately reflect current clinical practice and offer utility to inform recommended guidelines. Additionally, the rarity of minimally displaced FNF does not afford the opportunity to solely include high volume experienced surgeons [27]. We used a “sample to redundancy” approach by which we solicited feedback from new orthopaedic surgeons until no new items for the questionnaire emerged. An independent pretest evaluated face and content validity, ensuring the survey addressed current practice and study objectives. These orthopaedic surgeons also commented on the clarity and comprehensiveness of the questionnaire.

### Survey description

The survey consisted of 24 questions, including both multiple-choice and short open-ended questions. The survey was divided in four sections including demographic questions, management of minimally displaced FNFs, clinical vignettes, and trial participation (Online Appendix A).

**Fig. 1:**
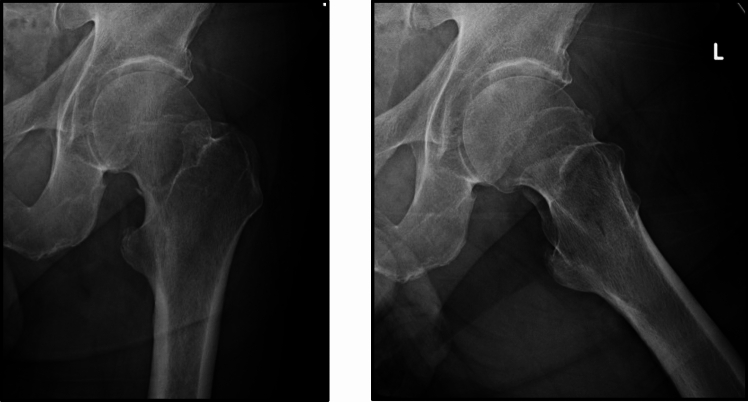
Radiographs of a non-displaced femoral neck fracture

**Fig. 2:**
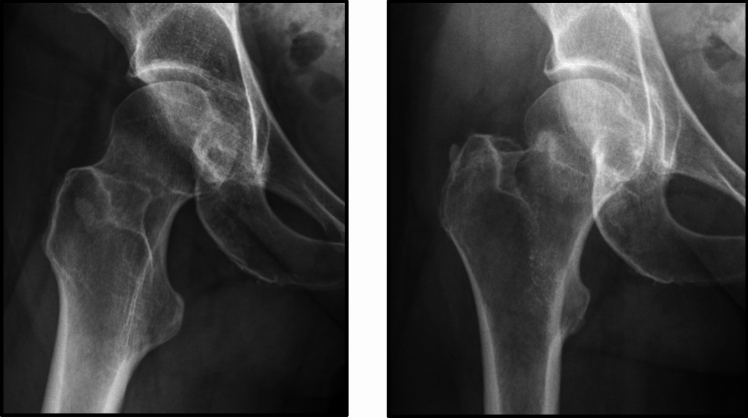
Radiographs of a valgus impacted femoral neck fracture

**Fig. 3:**
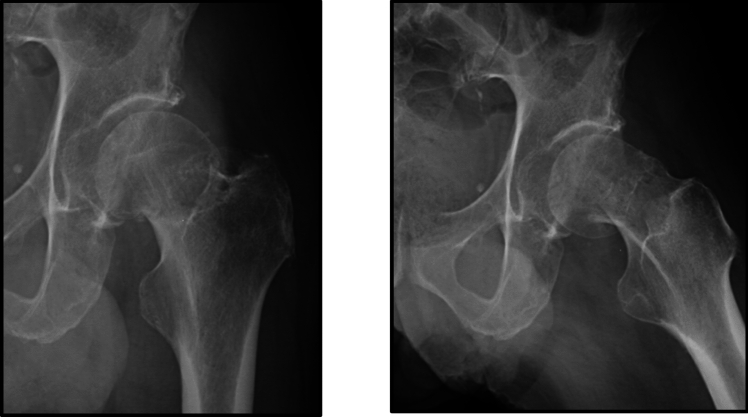
Radiographs of a posterior tilt between 15º and 20º

Survey respondents were provided three radiographic FNF scenarios and asked if they would be willing to randomize four hypothetical participants into a trial comparing arthroplasty versus IF. The radiographs included a non-displaced FNF (Fig. 1), a valgus impacted FNF (Fig. 2), and posterior tilt between 15° and 20° (Fig. 3). The four patients included: (1) an 80 year-old female, ASA III, low demand, who lives independently at home; (2) a 65 year-old female, ASA I, healthy and active; (3) a 90 year-old female, ASA IV, who uses a walker; and (4) a 90 year-old female with dementia. Respondents unwilling to randomize the participant were asked if they would treat the hypothetical trial participant with arthroplasty, either hemiarthroplasty or total hip arthroplasty per the preference of the treating surgeon, or IF. Lastly, survey respondents were also asked to grade the need and their interest in a definitive clinical trial comparing arthroplasty versus IF in minimally displaced FNFs.

### Survey administration

After obtaining approval from the Hamilton Integrated Research Ethics Board (HiREB #15740), the Canadian Orthopaedic Association (COA) and Orthopaedic Trauma Association (OTA) informed their members about the questionnaire via email and by posting the survey link to their website. To increase the response rate, the survey link was also sent to the investigators’ professional contacts via email. The completion of the survey was voluntary and involved no monetary incentive. The survey was distributed to 627 surgeons in January and February 2023.

### Sample size

To determine the number of respondents needed to ensure sufficient precision in our analysis, we applied a conservative estimate based on recent data that 90% of surgeons would prefer to treat minimally displaced FNFs with IF [16]. Assuming a 95% confidence interval for preference estimates with *α* = 0.05, at least 138 completed questionnaires would be necessary.

### Statistical analysis

All survey responses were analysed using descriptive statistics reported as count (per cent) for dichotomous and categorical variables. Chi-square analyses were performed to assess the association between 1) the preference for arthroplasty and the number of years in practice (> 10 years of practice versus < 10 years) and 2) the preference for arthroplasty and the number of minimally displaced FNFs treated annually (> 20 fractures versus < 20 fractures). All analyses were conducted in R (version 4.1.3, R Foundation for Statistical Computing, Vienna, Austria).

### Participant demographics

One hundred and fifty-five orthopaedic surgeons completed the demographics portion of the survey, with approximately half (45%) practising outside North America (Table 1). Sixty-five per cent had more than ten years of experience treating femoral neck fractures. Sixty-five per cent reported treating more than 30 FNF (both displaced and minimally displaced annually), but 88% reported treating 20 or less *minimally displaced* FNF annually. Fifty-six per cent worked in a facility where trainees, such as fellows and residents, were involved in the surgical treatment of these patients.

**Table 1:** Demographics

	N (%) N = 155
Region, n (%)	
USA	47 (30.3%)
Canada	38 (24.5%)
South America	28 (18.1%)
Europe	19 (12.3%)
Australia & New Zealand	13 (8.4%)
Africa	10 (6.5%)
Asia	0 (0.0%)
Experience treating femoral neck fractures, n (%)	
Less than 5 years	23 (14.8%)
5 to 10 years	32 (20.6%)
Greater than 10 years	100 (64.5%)
Number of femoral neck fractures treated annually, n (%)	
Less than 30	55 (35.5%)
30 to 50	70 (45.2%)
Greater than 50	30 (19.4%)
Number of minimally displaced femoral neck fractures treated annually, n (%)	
Less than 10	70 (45.2%)
10 to 20	67 (43.2%)
Greater than 20	18 (11.6%)
Trainees participating in surgical care of hip fractures, n (%)	
Yes	
Fellows and residents	86 (55.5%)
Fellows only	3 (1.9%)
Residents only	42 (27.1%)
No	24 (15.5%)

### Management of minimally displaced FNFs: IF vs. arthroplasty

One hundred and fifty-five orthopaedic surgeons completed the portion of the survey related to management of low-energy minimally displaced FNFs. Seventy-four per cent reported performing IF in at least half of minimally displaced FNFs, whereas 36% of surgeons reported performing arthroplasty in at least half of minimally displaced FNFs. Seventy-four per cent indicated that deciding between IF and arthroplasty is difficult for certain minimally displaced FNFs cases (Table 2).

**Table 2:** Management of Low-Energy Minimally Displaced Femoral Neck Fractures

	N (%) N = 155
Frequency of internal fixation in minimally displaced femoral neck fractures, n (%)	
Rarely	18 (11.6%)
Occasionally	23 (14.9%)
Half of the time	18 (11.6%)
Frequently	60 (38.7%)
Very frequently	36 (23.2%)
Frequency of arthroplasty in minimally displaced femoral neck fractures, n (%)	
Rarely	46 (29.7%)
Occasionally	53 (34.2%)
Half of the time	20 (12.9%)
Frequently	24 (15.5%)
Very frequently	12 (7.7%)
Difficulties in deciding between the two treatments for minimally displaced femoral neck fractures, n (%)	
Strongly agree	35 (22.6%)
Agree	79 (51.0%)
Neutral	15 (9.7%)
Disagree	21 (13.5%)
Strongly disagree	5 (3.2%)
Patient characteristics influencing treatment choice for minimally-displaced femoral neck fractures, n (%)	
Age	129 (83.2%)
Pre-injury mobility or walking aids	117 (75.5%)
Osteoporosis	96 (61.9%)
Independence with activities of daily living	94 (60.7%)
Neuromuscular disorder (Parkinson’s disease, post-polio syndrome, etc.)	88 (56.8%)
Dementia	70 (45.2%)
Frailty Index	56 (36.1%)
Body mass index	40 (25.8%)
Other	38 (24.5%)
Sex	19 (12.3%)

Patient characteristics influencing surgeons’ treatment preferences included: age (83%), pre-injury mobility or use of walking aids (76%), osteoporosis (62%), independence with activities of daily living (61%), neuromuscular disorder (57%), and dementia (45%) (Table 2). There was a statistically significant association between the preference for arthroplasty and the number of minimally displaced FNFs treated annually (*p* = 0.033), but none between arthroplasty preference and the number of years in practice (*p* = 0.065).

### X-ray parameters influencing treatment choice

One hundred and fifty-five orthopaedic surgeons completed the portion of the survey inquiring about what x-ray parameters influence their choice in treatment. Most surgeons indicated that they would perform arthroplasty for a posterior tilt of 20° (69%) or 30° (94%), varus angulation (88%), or a neck-shaft angle > 160° (70%). Conversely, IF was preferred for a posterior tilt of < 10° (83%) or a neck-shaft angle between 140° and 150° (83%) **(**Table 3**)**.

**Table 3:** X-ray Parameters Influencing Treatment Choice

	Internal Fixation N (%) N = 155	Arthroplasty N (%) N = 155
Posterior tilt 10º, n (%)	129 (83.2%)	26 (16.8%)
Posterior tilt 20º, n (%)	48 (31.0%)	107 (69.0%)
Posterior tilt 30º, n (%)	10 (6.5%)	145 (93.5%)
Varus, n (%)	18 (11.6%)	137 (88.4%)
Neck-shaft angle > 140º, n (%)	128 (82.6%)	27 (17.4%)
Neck-shaft angle > 150º, n (%)	84 (54.2%)	71 (45.8%)
Neck-shaft angle > 160º, n (%)	46 (29.7%)	109 (70.3%)

### Clinical vignettes

One hundred and fifty orthopaedic surgeons of the 155 (97%) completed the portion of the survey related to the three clinical vignettes: (1) non-displaced FNF (Fig. 1), (2) a valgus impacted FNF (Fig. 2), and (3) a FNF with a posterior tilt between 15º and 20º (Fig. 3).

The clinical scenarios demonstrated a wide variation in practice and treatment of low-energy minimally displaced FNF patients (Table 4). The responses suggest that respondents feel less comfortable with randomizing an active 65-year-old healthy female patients with no fracture angulation to either IF or arthroplasty: only 39% of respondents would randomize the patient, and 9% would perform only arthroplasty in this patient scenario in the absence of posterior fracture tilt. We also found that valgus fracture angulation did not seem to influence orthopaedic surgeons’ decision in treatment choice as nearly half of surgeons would select either procedure in most cases. In cases with posterior tilt, arthroplasty was the preferred treatment indicated by most surgeons (approximately 60% on average). Dementia did not seem to impact orthopaedic surgeons’ decision in treatment choice as nearly half of surgeons would select either procedure. X-ray parameters have a smaller influence on the treatment decision in older patients than in younger patients.

**Table 4:** Clinical Scenarios: Participant’s Willingness to Randomize to Internal Fixation Versus Arthroplasty

	Scenario #1 No coronal or sagittal fracture angulation N (%) N = 150*	Scenario #2 Valgus angulation, no sagittal fracture angulation N (%) N = 150	Scenario #3 No coronal fracture angulation, 10° posterior tilt N (%) N = 150*
80-year-old female, low demand, independent at home and ASAIII, n (%)			
Yes	71 (47.3%)	79 (52.7%)	39 (26.0%)
No, only IF	48 (32.0%)	44 (29.3%)	15 (10.0%)
No, only arthroplasty	31 (20.7%)	27 (18.0%)	96 (64.0%)
65-year-old female, healthy and active, n (%)			
Yes	59 (39.3%)	68 (45.3%)	50 (33.3%)
No, only IF	78 (52.0%)	66 (44.0%)	30 (20.0%)
No, only arthroplasty	13 (8.7%)	16 (10.7%)	70 (46.7%)
90-year-old female, walker and ASA IV, n (%)			
Yes	59 (39.3%)	59 (39.3%)	38 (25.3%)
No, only IF	46 (30.7%)	41 (27.3%)	16 (10.7%)
No, only arthroplasty	45 (30.0%)	50 (33.3%)	96 (64.0%)
90-year-old female with dementia, n (%)			
Yes	59 (39.3%)	53 (35.3%)	36 (24.0%)
No, only IF	49 (32.7%)	47 (31.3%)	18 (12.0%)
No, only arthroplasty	42 (28.0%)	50 (33.3%)	96 (64.0%)

^*^5 respondents did not complete this portion of the survey

### Need for a clinical trial

In all scenarios, at least one-third of surgeons were willing to randomize similar patients to receive either arthroplasty or IF. Seventy-nine per cent of surgeons agreed that a randomized controlled trial is needed to compare arthroplasty to IF for minimally displaced FNFs and 71% of respondents would be willing to participate in the trial (Table 5).

**Table 5:** Need for a Trial

	N (%) N = 155
Need to conduct a randomized controlled trial, n (%)	
Strongly agree	61 (40.7%)
Agree	58 (38.6%)
Neutral	16 (10.7%)
Disagree	13 (8.6%)
Strongly disagree	2 (1.4%)
Willing to participate in the randomized controlled trial, n (%)	
Yes, I would like to participate	107 (71.3%)
Unsure	20 (13.4%)
No, not at this time	23 (15.3%)

## Discussion

This study found a lack of agreement among orthopaedic surgeons in the management of minimally displaced FNFs. The variation between orthopaedic surgeon’s current practices suggests the need for a high-quality randomized trial to definitively address this patient important question. This interpretation was similarly conveyed by the majority of respondents endorsing the need for a randomized controlled trial.

Surgeon preferences between internal fixation and arthroplasty for older adult patients are based more on perceptions of risk and historical practice than evidence [8]. The strength of the AAOS 2021 Clinical Practice Guideline recommendation for internal fixation of minimally displaced femoral neck fractures was downgraded from prior versions because the evidence to support this recommendation is limited [10, 31]. The AAOS Evidence-Based Quality and Value Committee responsible has indicated that their guidelines would benefit from higher quality data (*personal communication*). Current practice guidelines from the National Institute for Health and Care Excellence in the UK also cite a lack of sufficient evidence to recommend treatment [11], while those from the European Society of Trauma and Emergency Surgery [12], Australian and New Zealand Hip Fracture Registry [32], and Japanese Orthopaedic Association [33] advocate for internal fixation despite emerging evidence to support arthroplasty [12, 32, 33].

We sought to determine if practising surgeons would have equipoise to randomize patients with minimally displaced FNFs to IF or arthroplasty. We pursued this by querying surgeons about their practice habits. We also sought to identify regional, surgeon, patient, and fracture characteristics associated with the clinical decision making. We attempted to elucidate these through direct questions and clinical vignettes. In contrast to existing guidelines and prior practice trends, we observed that over one-third of orthopaedic surgeons who responded to the questionnaire routinely treat minimally displaced FNFs with arthroplasty. While IF is the preferred method of treatment for minimally displaced FNFs, the majority of respondents agreed there are difficulties in deciding between IF and arthroplasty when treating patients. When presented with different clinical scenarios, in nearly all cases, at least one-third of surgeons were willing to randomize similar patients to receive either arthroplasty or IF. This suggests surgeon equipoise for a randomized controlled trial. In addition, respondents agreed on the need for a clinical trial.

Two recent systematic reviews and one clinical trial suggest that IF for minimally displaced FNFs is associated with higher rates of complications and reoperations than arthroplasty. Overman et al. [13] focused on the outcomes of IF patients and reported a high complication rate for non-displaced FNFs treated with IF, with a risk of reoperation and mortality exceeding 14%. Richards et al. concluded that hemiarthroplasty may reduce the risk of reoperation by 70% when compared with IF [23]. Dolatowski et al. [3] randomly allocated 219 Norwegian patients to IF or hemiarthroplasty procedures and found that hemiarthroplasty improved mobility and with fewer major reoperations, although that study was not sufficiently powered to detect a difference in mortality. This emerging literature is compelling but does not provide sufficient evidence to support a widespread change in practice. A randomized clinical trial would provide this evidence.

Patients and surgeons must have equipoise between treatments to participate in such a trial. We found that surgeons’ treatment preferences were most influenced by patient age, pre-injury mobility or use of walking aids, and osteoporosis. A systematic review and meta-analysis identified that failure of IF in displaced FNFs was associated with sex (females), age (over 50 years old), and smoking habits [17]. A case-cohort study performed by Gregersen et al. [34] found reoperations in the IF study group were associated with lower age and status of independent living, which they hypothesized could be related to the greater physical function. Our survey found that three out of four surgeons considered pre-injury mobility or walking aids and independence with activities of daily living (61%) in their treatment decision. Clement et al. [18] identified ASA grade as a predictor of fixation failure and mortality following IF for minimally displaced FNFs. Although our survey did not include ASA classification as an option for factors that influenced treatment decision, researchers did introduce the Frailty Index, which also measures comorbidities. However, only 36% of the respondents reported that frailty influenced their decision.

According to the results of the survey, most surgeons would perform arthroplasty when the posterior tilt is greater than 20°. Kalsbeek et al. [19] reported a failure ratio 4 times higher in Garden I and II FNFs treated with dynamic locking blade plate when the posterior tilt is ≥ 20° [35]. In another retrospective study of over 1500 patients with the same Garden-type fractures but treated with 2 pins or 2–3 cannulated screws, a posterior tilt over 20° was seen to be a risk factor to failure and reoperations. Biz et al. [36] showed that Garden type II, Pauwels II and III, and a posterior tilt > 18° were predictors of early failures. Okike et al. [37], Dolatowski et al. [38], Sjöholm et al. [39], and Nielsen et al. [40] also support the finding of an increased failure rate when the posterior tilt is > 20°. However, one study reported no difference in failure rate among posterior tilts [41].

This study has limitations. Selection and response bias may result from our convenience sample of researcher contacts, society distribution to North American surgeons, and our response rate of 25%. However, this work is strengthened by a thorough survey development process, responses from an international cohort of surgeons, and the use of multiple question types including multiple choice, open ended, radiograph- and scenario-based questions to engage survey respondents and identify factors influencing treatment choices. The radiographic images depicted in the survey include rotated, poor-quality lateral radiographs intended to reflect suboptimal clinical information from which treatment data decisions are made in real-world practice. In some circumstances, computed tomography may be helpful for better classification and treatment of these injuries.

In conclusion, this international survey of orthopaedic surgeons identified a lack of consensus among surgeons regarding the optimal surgical management of minimally displaced FNFs but did identify specific clinical scenarios in which treatment decisions become more clearly defined. Additionally, the survey revealed the surgeons’ agreement on the need for a high-quality randomized trial to definitively determine if arthroplasty or IF leads to better patient outcomes. Clinical trials to address this clinical question including Fixation Versus Arthroplasty Surgical Treatments for Early Recovery after HIP fracture (FASTER-HIP), World Hip Trauma Evaluation 11 - Fix or Replace Undisplaced Intracapsular fractures Trial of Interventions (FRUITI) [42], and Hips Screws or (Total) Hip Replacement for Undisplaced Femoral Neck Fractures in Elderly Patients (HipSTHeR) [43] are actively enrolling participants.

## Supplementary Information

Below is the link to the electronic supplementary material.

Supplementary file1 (DOCX 980 KB)

## Data Availability

Data may be available upon request.
