# Supplementary material for: When would orthopaedic surgeons perform arthroplasty for a femoral neck fracture in an older adult?
Source: Eur J Orthop Surg Traumatol. 2025 Jul 11;35(1):301. doi: 10.1007/s00590-025-04412-3 (PMC12254171; doi:10.1007/s00590-025-04412-3)
Supplement: Supplementary file 1 — Supplementary file1 (DOCX 980 KB) [file 590_2025_4412_MOESM1_ESM.docx]

**Supplemental Digital Content 1: Survey**

Minimally displaced femoral neck fractures have traditionally been treated with internal fixation because it is minimally invasive, and therefore believed to reduce the risk of complications and mortality. However, there is increasing evidence that internal fixation may not be the optimal treatment option for these fractures. New data suggests primary arthroplasty could improve patient outcomes by reducing the risk of major reoperations, decreasing mortality, and improving mobility. We believe that clinical practice would benefit from a definitive randomized controlled trial that clearly defines the optimal surgical treatment for patients with minimally displaced femoral neck fractures.

Thank you for participating in our survey. Your feedback is important.

Part 1: Demographic Questions:

1. Where are you currently practicing? (Check one)

□ Canada □ USA □ Europe □ Asia □ South America

□ Australia & New Zealand □ Africa

2. For how many years have you been treating femoral neck fracture patients? (Check one)

□ Less than 5 years

□ 5 to 10 years

□ Greater than 10 years

3. Approximately how many low energy (fragility) femoral neck fractures do you treat on an annual basis? (Check one)

□ Less than 30

□ 30 to 50

□ Greater than 50

4. Approximately how many low energy (fragility) minimally displaced femoral neck fractures do you treat on an annual basis? (Check one)

□ Less than 10

□ 10 to 20

□ Greater than 20

5. Do trainees routinely participate in surgical care of hip fractures at your institution?

□ Yes, fellows and residents

□ Yes, fellows only

□ Yes, residents only

□ No

Feel free to add any comment on your choices above:

Part 2: Management of Low Energy Minimally displaced Femoral Neck Fractures

The following questions ask about your practice patterns regarding the surgical management of the low energy (fragility) minimally displaced femoral neck fractures.

1. How often do you perform internal fixation in patients with minimally displaced femoral neck fractures? (Check one)

Rarely

Occasionally

Half of the time

Frequently

Very frequently

2. How often do you perform arthroplasty in patients with minimally displaced femoral neck fractures? (Check one)

Rarely

Occasionally

Half of the time

Frequently

Very frequently

3. In managing minimally displaced femoral neck fractures, there are some cases in which it is difficult to decide between internal fixation and arthroplasty as the definitive fracture management.

Strongly agree

Agree

Neutral

Disagree

Strongly disagree

4. Which of the following patient characteristics influence your treatment choice for minimally displaced femoral neck fractures? (Select all that apply)

Age

Sex

Pre-injury mobility or walking aids

Independence with activities of daily living

Neuromuscular disorder (Parkinson’s disease, post-polio syndrome, etc.)

Dementia

Frailty Index

Osteoporosis

Body mass index

Other, specify: ____________________________________________

5. Please indicate which procedure you would perform (internal fixation or arthroplasty) for each of the radiographic characteristics below (assuming the patient is a candidate for both procedures):

| **Radiographic Characteristic** | **Which treatment do you prefer?** |
| --- | --- |
| 1. Posterior tilt 10º | ☐ Internal Fixation  ☐ Arthroplasty |
| 1. Posterior tilt 20º | ☐ Internal Fixation  ☐ Arthroplasty |
| 1. Posterior tilt 30º | ☐ Internal Fixation  ☐ Arthroplasty |
| 1. Varus | ☐ Internal Fixation  ☐ Arthroplasty |
| 1. Neck-shaft angle >140º | ☐ Internal Fixation  ☐ Arthroplasty |
| 1. Neck-shaft angle >150º | ☐ Internal Fixation  ☐ Arthroplasty |
| 1. Neck-shaft angle >160º | ☐ Internal Fixation  ☐ Arthroplasty |

Feel free to add any comment on your choices above:

Part 3. Trial Specific Questions

We would like your input on three different patient scenarios to help inform the design of this trial. Please review the x-rays and each clinical scenario and indicate if you would randomize the patient into a trial comparing internal fixation and arthroplasty.

**Scenario #1:**

| 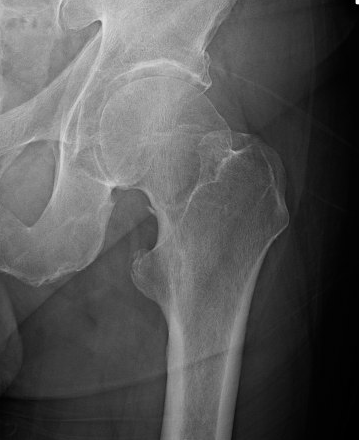 | 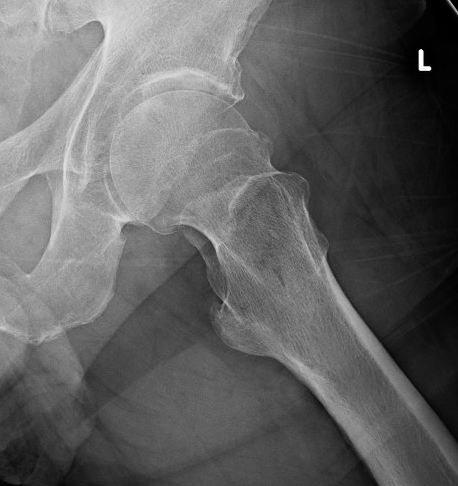 |
| --- | --- |

1. This is an 80 year-old female, low demand, lives independently at home, and ASA III. Would you randomize this patient?

Yes

No, I would only perform internal fixation

No, I would only perform arthroplasty

2. This is a 65 year-old female who is healthy and active. Would you randomize this patient?

Yes

No, I would only perform internal fixation

No, I would only perform arthroplasty

3. This is a 90 year-old female, ASA IV, and uses a walker. Would you randomize this patient?

Yes

No, I would only perform internal fixation

No, I would only perform arthroplasty

4. This is a 90 year-old female with dementia. Would you randomize this patient?

Yes

No, I would only perform internal fixation

No, I would only perform arthroplasty

Feel free to add any comment on your choices above:

**Scenario #2:**

| 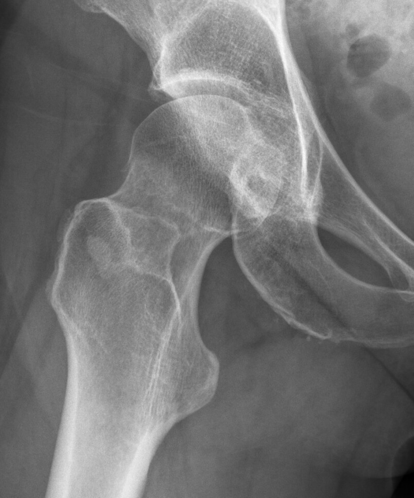 | 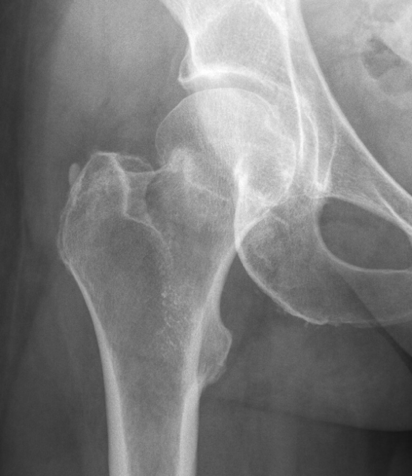 |
| --- | --- |

5. This is an 80 year-old female, low demand, lives independently at home, and ASA III. Would you randomize this patient?

Yes

No, I would only perform internal fixation

No, I would only perform arthroplasty

6. This is a 65 year-old female who is healthy and active. Would you randomize this patient?

Yes

No, I would only perform internal fixation

No, I would only perform arthroplasty

7. This is a 90 year-old female, ASA IV, and uses a walker. Would you randomize this patient?

Yes

No, I would only perform internal fixation

No, I would only perform arthroplasty

8. This is a 90 year-old female with dementia. Would you randomize this patient?

Yes

No, I would only perform Internal Fixation

No, I would only perform Arthroplasty

Feel free to add any comment on your choices above:

**Scenario #3:**

| 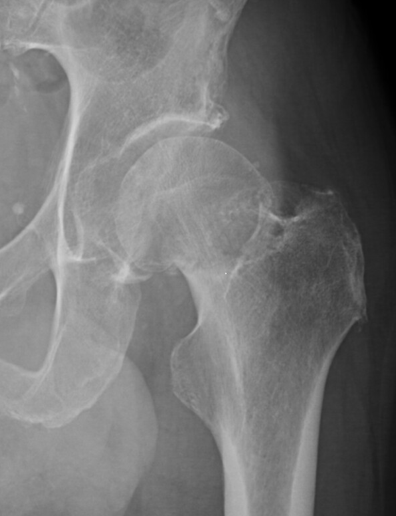 | 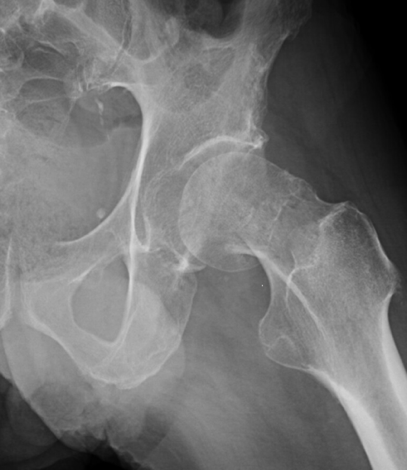 |
| --- | --- |

9. This is an 80 year-old female, low demand, lives independently at home, and ASA III. Would you randomize this patient?

Yes

No, I would only perform internal fixation

No, I would only perform arthroplasty

10. This is a 65 year-old female who is healthy and active. Would you randomize this patient?

Yes

No, I would only perform internal fixation

No, I would only perform arthroplasty

11. This is a 90 year-old female, ASA IV, and uses a walker. Would you randomize this patient?

Yes

No, I would only perform internal fixation

No, I would only perform arthroplasty

12. This is a 90 year-old female with dementia. Would you randomize this patient?

Yes

No, I would only perform internal fixation

No, I would only perform arthroplasty

Feel free to add any comment on your choices above:

Part 4: Trial Participation

1. There is a need in the surgical community to conduct a randomized controlled trial comparing internal fixation and arthroplasty in minimally displaced femoral neck fracture:

Strongly agree

Agree

Neutral

Disagree

Strongly disagree

2. Would you be willing to participate in a randomized controlled trial internal fixation and arthroplasty for minimally displaced femoral neck fractures to assess that knowledge gap?

Yes, I would like to participate

Unsure

No, not at this time

3. Please provide any comments or additional information:

Thank you for having completed the Survey

If you want to receive further communications regarding this study, write an email to XXXXXX.ca
